# Effect of fetal calf serum on propagation of duck hepatitis A virus genotype 3 in duck embryo fibroblast cells

**DOI:** 10.1186/s12917-019-1904-y

**Published:** 2019-05-17

**Authors:** Minghang Wang, Ziheng Li, Huicong Liu, Xiaoyan Wang, Dabing Zhang

**Affiliations:** Key Laboratory of Animal Epidemiology of the Ministry of Agriculture, College of Veterinary Medicine, China Agricultural University, No. 2 Yuanmingyuan West Road, Haidian district, Beijing, 100193 People’s Republic of China

**Keywords:** Duck hepatitis a virus genotype 3, Duck embryo fibroblast cells, Fetal calf serum, Inhibit

## Abstract

**Background:**

Duck viral hepatitis (DVH) is a highly contagious viral disease affecting ducks. It can be caused by five agents, including duck hepatitis A virus genotypes 1 (DHAV-1), 2 (DHAV-2), and 3 (DHAV-3), as well as duck hepatitis virus 2 and duck hepatitis virus 3. Since 2007, DHAV-3 has been known to be the most prevalent in East and South Asia. So far, the information regarding the propagation of DHAV-3 in cultured cells is limited. In this study, we describe the comparative studies on the growth properties of DHAV-3 in primary duck embryo fibroblast (DEF) cells using two different strains: a virulent strain C-GY and an attenuated strain YDF120. The effect of fetal calf serum (FCS) and chick serum (CS) on DHAV-3 replication and the mechanism of the inhibitory effect conferred by FCS were also investigated.

**Results:**

Following serial passages, both C-GY and YDF120 failed to produce cytopathic effect and plaques. The combined quantitative real-time PCR and indirect immunofluorescence staining methods showed that the two viruses could be propagated productively in DEF cells. Investigation of the viral growth kinetics revealed that the two viruses replicated in DEF cells with similar efficiencies, while the viral load of the virulent C-GY strain peaked more rapidly when compared with the attenuated YDF120 strain. Neutralization assay and time-of-drug-addition study indicated that FCS displayed inhibitory effect on DHAV-3 replication. Analysis on the mechanism of action of FCS against DHAV-3 demonstrated that the inhibitory effect was reflected at three steps of the DHAV-3 life cycle including adsorption, replication, and release.

**Conclusions:**

Both virulent and attenuated DAHV-3 strains can establish noncytocidal, productive infections in DEF cells. The virulent strain replicates more rapidly than the attenuated strain in early infection period. FCS has an inhibitory effect on DHAV-3 replication, which may be attributed to action of a non-specific inhibitory factor present in FCS directly on the virus. These findings may provide new insights into the development of potential antiviral agents.

## Study design

Since the growth of duck hepatitis A virus (DHAV) in cultured cells is curial to explore the molecular mechanisms involved in replication and pathogenesis of the virus, we investigated comparative studies on the growth properties of two different DHAV-3 strains (virulent isolate C-GY from duck viral hepatitis (DVH) of Pekin ducklings and attenuated strain YDF120) in primary duck embryo fibroblast (DEF) cell cultures and the effect of fetal calf serum (FCS) and chick serum (CS) on DHAV-3 replication. The study design of this article are as follows:

1. Culture the DHAV-3 C-GY and YDF120 strains in primary DEF cells and passage the viruses for 10 times and monitor the formation of cytopathic effect (CPE) and plaque in cells.

2. Investigate the expression of viral proteins in DEF cells inoculated with DHAV-3 by using an indirect immunofluorescence assay, employing specific chicken antisera against DHAV-3 C-GY and a FITC-conjugated goat anti-chicken IgG.

3. Detect the viral loads in each passage of C-GY and YDF120 by using a DHAV-3 quantitative real-time PCR (qPCR) assay.

4. Investigate and compare the replication kinetics of DHAV-3 virulent and attenuated strains in DEF cultures via detection of viral loads of the 5th cell passages of C-GY and YDF120 using the DHAV-3 qPCR assay. The 5th cell passages of C-GY and YDF120 were also employed in the subsequent experiments.

5. Assess the effect of FCS and CS on virulent and attenuated strains of DHAV-3 in DEF cells by using neutralization tests.

6. Investigate the mode of action of FCS on DHAV-3 replication in DEF cells by using time-of-drug-addition assay.

7. Examine the inhibitory effect on the steps of the DHAV-3 life cycle by using four different experiments.

## Background

Duck viral hepatitis (DVH) is an acute and highly fatal contagious disease of ducklings, characterized primarily by hepatitis [1]. It can be caused by five viruses. They include duck hepatitis A virus genotypes 1 (DHAV-1; formerly duck hepatitis virus type 1) [2], 2 (DHAV-2) [3], and 3 (DHAV-3) [4], members of the species *Avihepatovirus A* of the genus *Avihepatovirus* in the family *Picornaviridae* [5] (http://www.picornaviridae.com/avihepatovirus/avihepatovirus.htm), and duck hepatitis virus type 2 (DHV-2) [6–8] and duck hepatitis virus type 3 (DHV-3) [9], which are currently classified within the genus *Astrovirus* in the family *Astroviridae* [10, 11]. Compared with DHV-2 and DHV-3, the three DHAV genotypes can cause more severe diseases [1, 3, 12, 13]. Among the DHAV genotypes, DHAV-1 is known to be worldwide in distribution [1]; DHAV-2 has only been reported in Taiwan, China [3]; and DHAV-3 has been found in South Korea [4], mainland China [14], and Vietnam [15]. Since its first report in 2007, DHAV-3 has been known to be the most prevalent in duck industry in East and South Asia [4, 14–18].

The genome sequence has been determined for a number of DHAV isolates, as indicated in GenBank records. The genome of DHAVs consists of positive-sense, single-stranded, polyadenylated RNA of 7689–7775 nucleotides (nt). The polyadenylated genome contains a large open reading frame (ORF), encoding a putative polyprotein, which is flanked by the 5′ and 3′ untranslated regions (UTRs). The DHAV polyprotein appears to be cleaved into three structural (VP0, VP3 and VP1) and 8–9 nonstructural (2A1, 2A2 [or 2A2, 2A3], 2B, 2C, 3A, 3B, 3C and 3D) proteins [3, 4, 19–21]. Comparative sequence analysis demonstrates that DHAV-3 strains share low identity at the nucleotide (genome: 70–73%) and amino acid (polyprotein: 82–83%) level with DHAV-1 strains [4, 12, 22]. Moreover, DHAV-3 and DHAV-1 differ greatly in their genome lengths [4, 12, 19–21]. Previous works have shown that DHAV-3 has no common antigens with DHAV-1 in virus neutralization tests [4, 14]. Thus, emergence of DHAV-3 in Asia has caused great concern for duck industry.

The growth of DHAV in cultured cells is curial to explore the molecular mechanisms involved in replication and pathogenesis of the virus. Because DHAV-1 is the most common virus reported in most outbreaks worldwide, most data regarding to the growth of DHAV in cell cultures was obtained from studies on DHAV-1. Attempts to propagate DHAV-1 in cell cultures of avian embryo origin have been reported [20, 23–39]. However, cytopathic effect (CPE) was only observed in some types of cell cultures following infection with DHAV-1. In addition, conflicting results were obtained from different researchers. For example, Hwang concluded that duck embryo liver (DEL) cells were unsuitable for the propagation of DHAV-1 [29], whereas Woolcock reported that DHAV-1 was propagated successfully in DEL cells [39]. The study by Davis and Woolcock showed that attenuated DHAV-1 grew in embryo cell cultures of goose, turkey, quail, pheasant, guinea fowl, and chicken origin, while virulent virus strains grew to varying degrees in only guinea fowl, quail, and turkey embryo cells, supporting the view that virulent and attenuated DHAV-1 may exhibit difference in terms of growth property [24]. So far, there is only one report describing the propagation of DHAV-3 in DEL cells, and DHAV-3 behaved similarly to DHAV-1 with respect to induction of CPE in virus-infected cells [39, 40].

Plaque assays in duck embryo kidney (DEK) and DEL cell monolayers have demonstrated that mammalian sera can inhibit the growth of DHAV-1 [38, 39, 41]. In the study by Woolcock et al., a plaque assay was developed and applied for the assay of attenuated DHAV-1 in DEK cells, which showed that the concentration of fetal calf serum (FCS) in the overlay medium can affect both diameter and numbers of the plaques [38]. This observation was confirmed further by plaque assays for virulent and attenuated DHAV-1 in DEK and DEL cells [39]. The study by Chalmers and Woolcock demonstrated that several mammalian (e.g., fetal calf, newborn calf, rabbit and dog) sera had a drastic inhibitory effect on DHAV-1, and that the virus-inhibitory substance present in the sera appeared to act directly on the virus rather than preventing entry into the cells by blocking receptor sites [41]. As FCS are commonly used for virus propagation in cell cultures, the difficulty in growth of DHAV-1 in cell cultures might be attributed to the inhibitory effect of FCS on the virus. Whether or not FCS exhibits inhibitory effect on growth of DHAV-3 in cell cultures remains unknown.

In the present study, we describe the comparative studies on the growth properties of two different DHAV-3 strains (virulent isolate C-GY from DVH of Pekin ducklings and attenuated strain YDF120) in primary duck embryo fibroblast (DEF) cell cultures. and the effect of FCS and chick serum (CS) on DHAV-3 replication. The mechanism of the inhibitory effect of FCS against DHAV-3 replication was also investigated.

## Results

### DHAV-3 could establish noncytocidal infection in DEF cells

Initially, we propagated DHAV-3 in DEF cells using maintenance medium consisting of Dulbecco’s modified Eagle’s medium (DMEM) supplemented with 2% FCS. However, attempts to propagate the virus were unsuccessful. Thus, we used DMEM containing 2% CS as maintenance medium according to previously reported method to propagate DHAV-1 [38, 39, 41].

**Fig. 1:**
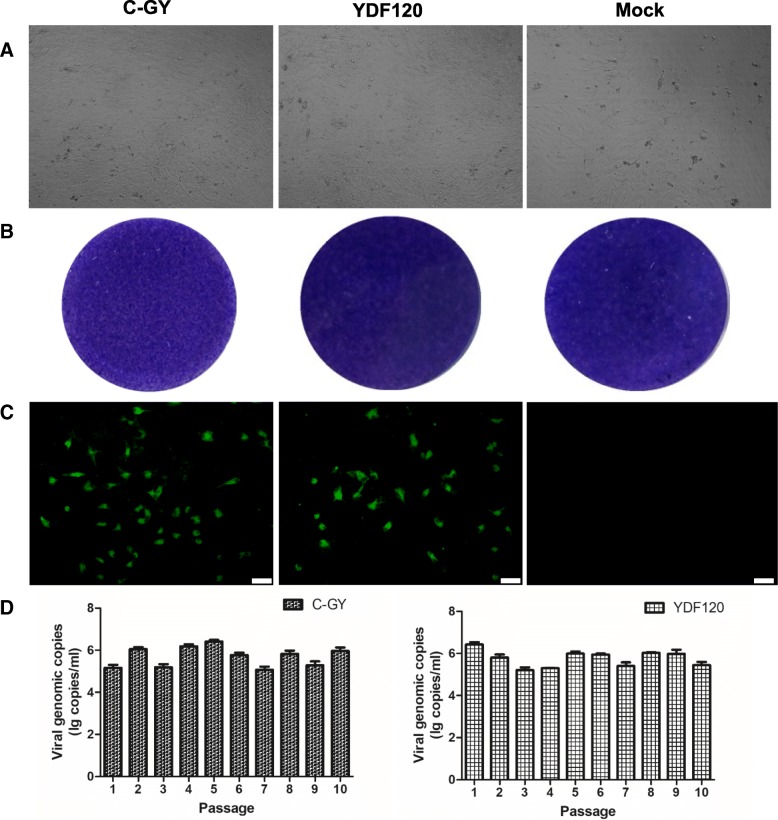
Characterization of growth of DHAV-3 in DEF cells. **a** Observation on formation of CPE in DEF cells. The picture was taken at 72 h after inoculation with the 10th passage of YDF120 and C-GY. **b** Assays on formation of plaques in DEF cells at 72 hpi. The 5th cell passages of DHAV-3 YDF120 and C-GY were used as inocula. The infected cells were stained with crystal violet, and viewed with direct light. **c** Analysis on expression of viral protein by immunostaining of DEF cells at 36 hpi. The 5th cell passages of DHAV-3 YDF120 and C-GY were employed to infect DEF cells. Viral protein expression was analyzed with specific chicken antisera against DHAV-3 C-GY and a FITC-conjugated goat anti-chicken serum (green). Bar = 50 μm. **d** Quantification of viral RNA copies in DEF cells at 72 hpi. RNA copies were detected by qPCR, and the results were shown for the first to tenth passages of DHAV-3 YDF120 and C-GY

The propagation of DHAV-3 in primary DEF cells was firstly tested by monitoring the formation of CPE and by plaque assays. Inoculation of DHAV-3 C-GY and YDF120 onto the DEF cells failed to induce CPE even after ten serial passages (Fig. 1a). There was no noticeable difference in cell morphology between the virus- and mock-infected DEF cells and between cells inoculated with different passages of C-GY and YDF120. In the plaque assays performed with the 5th passage of cell-derived viruses, both C-GY and YDF120 failed to form plaques (Fig. 1b).

An indirect immunofluorescence (IIF) assay was employed to characterize the growth of DHAV-3 in DEF cells. In this test, the cells 36 h post inoculation (hpi) with the 5th passages in DEF cells of DHAV-3 C-GY and YDF120 and the mock-infected cells were stained with fluorescein isothiocyanate (FITC)-conjugated goat anti-chicken IgG and specific chicken antisera to DHAV-3. As shown in Fig. 1c, the virus-infected cultures showed fluorescence, whereas no green fluorescent signals were observed in mock-infected cells. The investigations indicated that antigens of both viruses were expressed efficiently in DHAV-3-positive DEF cells.

To confirm further the growth of DHAV-3 in DEF cells, a DHAV-3 quantitative real-time PCR (qPCR) assay was used to quantify viral RNAs in samples (cells together with medium) harvested from the DHAV-3-infected cell cultures at 3 days post inoculation (dpi). The viral loads were determined to range from 10^5.1 ± 0.15^ to 10^6.4 ± 0.08^ copies/ml in cell cultures infected with the first to tenth passages of C-GY, and from 10^5.2 ± 0.12^ to 10^6.4 ± 0.11^ copies/ml in cell cultures infected with the first to tenth passages of YDF120 (Fig. 1d). The samples from the mock-infected cells were tested negative for DHAV-3.

### Virulent and attenuated DHAV-3 replicate with similar efficiencies

**Fig. 2:**
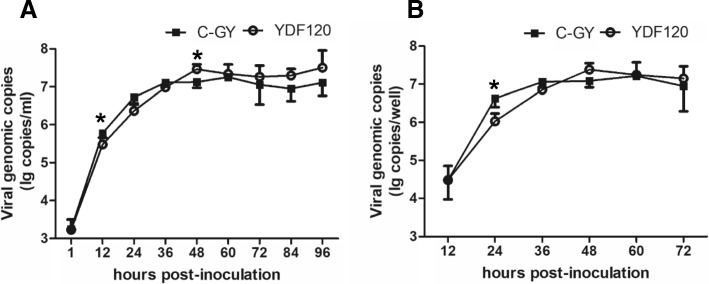
Growth kinetics of DHAV-3 in DEF cells. DEF cells were inoculated with the 5th cell passages of DHAV-3 YDF120 and C-GY at a MOI corresponding to 1 copy/cell. Viral yields in DEF cells were reflected by RNA copies detected with qPCR at indicated time after inoculation. At each time point, samples (medium together with cells) (**a**) and medium (**b**) were collected from three wells of 24-well-plates. Error bars represent the SD (*n* = 3). *, *P* < 0.05; **, *P* < 0.01

To investigate and compare the replication kinetics of C-GY and YDF120 in DEF cultures, the growth curves of DHAV-3 were established using the cell cultures (medium and cells) and medium. As shown in Fig. 2a and b, both C-GY and YDF120 could efficiently replicate in DEF cells. Differences in growth kinetics were detected between the two strains. Viral loads of C-GY and YDF120 peaked at 36 hpi and 48 hpi, respectively. The viral loads of C-GY were higher than those of YDF120 till C-GY reached the highest titer, afterwards the viral loads of YDF120 were higher than those of C-GY. Viral loads of C-GY and YDF120 differed significantly from each other in cell cultures at 12 and 48 hpi (*P* < 0.05) (Fig. 2a), and in medium at 24 hpi (*P* < 0.05) (Fig. 2b).

### FCS displayed inhibitory effect on DHAV-3 replication

**Fig. 3:**
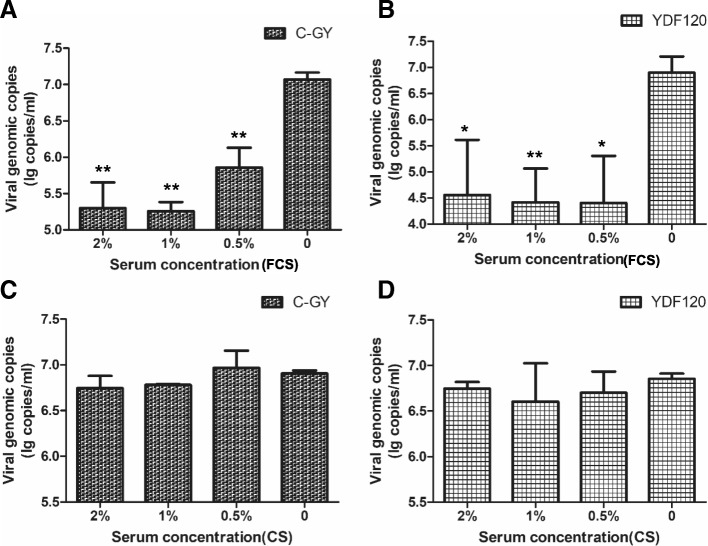
Effect of FCS and CS on DHAV-3 replication in DEF cells. (**a)** and (**b)** represent the effect of FCS on C-GY and YDF120 at different concentrations respectively. (**c)** and (**d)** represent the effect of CS on C-GY and YDF120 at different concentrations respectively. At each concentration, samples (medium and cells) were collected from three wells of 24-well-plates and viral load in each sample was quantified by qPCR. Error bars represent the SD (*n* = 3). *, *P* < 0.05. **, *P* < 0.01

To assess the effect of FCS and CS on DHAV-3 virulent and attenuated strains, neutralization tests were carried out. In these tests, each serum was diluted and mixed with an equal volume of virus to give three different final concentrations (2, 1 and 0.5%). A mixture of DMEM and an equal volume of virus served as control. The viral loads were determined at 36 hpi for C-GY and 48 hpi for YDF120 respectively. As shown in Fig. 3a and b, the vial loads of C-GY and YDF120 after treatment with different concentrations of FCS were shown to be significantly lower than those of control (*P* < 0.05). Compared with control, no significant difference was observed in viral loads of both C-GY and YDF120 after treatment with CS (*P* > 0.05) (Fig. 3c and d).

### FCS inhibited DHAV-3 replication at stages of co- and post-inoculation

**Fig. 4:**
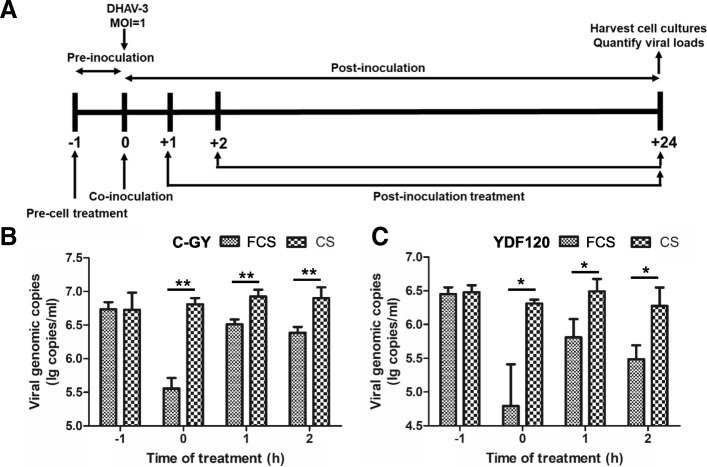
Analysis on the mode of action of FCS against the virulent and attenuated DHAV-3 strains. **a** Schematic illustration of the TODA assays. DEF cells were treated with FCS or CS at indicated time and were inoculated with C-GY or YDF120 (MOI of 1 copy/cell). (**b**) and (**c**) represent the results obtained from the TODA assays for C-GY and YDF120 respectively. At each time point, samples (medium and cells) were collected from three wells of 24-well-plates at 24 hpi and viral loads in samples were determined by qPCR assay. Error bars represent the SD (*n* = 3). *, *P <* 0.05; **, *P <* 0.01

To search the clue for the mode of action of FCS against the virulent and attenuated DHAV-3 strains, the time-of-drug-addition (TODA) assays were conducted (Fig. 4a), employing CS as a control. In the pre-cell treatment assays, there were no significant differences in the viral loads between FCS- and CS-treated groups (*P* > 0.05). In the co-inoculation studies, the viral loads detected in the FCS-treated group were significantly lower than those in the CS-treated group (*P* < 0.05). In the post-inoculation treatment assays, FCS remained inhibitory effect on replication of both C-GY and YDF, showing significant differences in viral loads when compared with the CS-treatment group (*P* < 0.05) (Fig. 4b and c).

### FCS inhibited DHAV-3 at various steps of the DHAV-3 life cycle

**Fig. 5:**
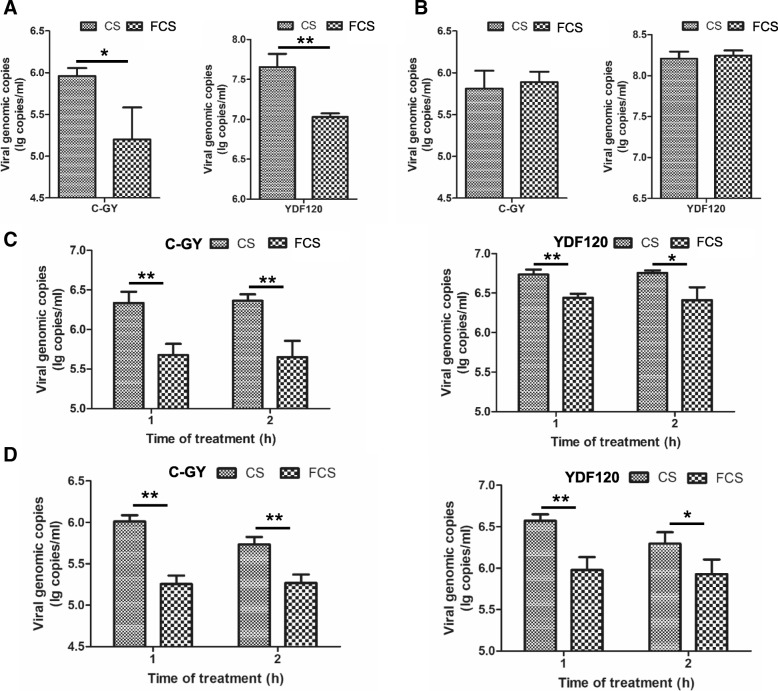
Examination of steps of the DHAV-3 life cycle on which FCS exhibited inhibitory effect. (**a**) Virus-serum mixtures were used as inoculation and incubated at 4 °C for 1 h to allow viral adsorption to cell surface. **b** Viral inoculation was performed at 4 °C for 1 h, followed by sera treatment at 37 °C. DHAV-3 inoculated cells were treated with sera at 1 h and 2 h after inoculation and the samples (medium together with cells) (**c**) and medium (**d**) were collected for further detection. All samples were collected from three well of 24-well-plates and the viral loads in samples were quantified by qPCR assay. Error bars represent the SD (*n* = 3). *, *P <* 0.05; **, *P <* 0.01

Viral attachment inhibition assay was performed to test the effect of FCS on adsorption of the C-GY and YDF120 viruses to cell receptor sites. In this test, virus-serum mixtures were used for inoculation and the cells were incubated at 4 °C for 1 h to allow viral adsorption to cell surface. As shown in Fig. 5a, the viral loads detected at 24 hpi for both C-GY and YDF120 in the FCS-treated group were significantly lower than those in the CS-treated group (*P* < 0.05), suggesting that pre-incubation of virus with FCS reduced significantly the replication efficiency of both C-GY and YDF120 in DEF cells.

To assess whether or not FCS can inhibit penetration of DHAV-3 into the interior of DEF cells, viral inoculation was performed at 4 °C for 1 h, followed by sera treatment at 37 °C. The viral loads derived from the FCS-treated group showed no significant difference when compared with those from the CS-treated group (*P* > 0.05) (Fig. 5b). The result indicated that FCS had no effect on entry of DHAV-3 into the cells.

As shown in Fig. 5c and d, addition of FCS at 1 h and 2 h after inoculation resulted in significant decreases in viral loads in both cell cultures (cells and medium) and medium, showing significant inhibitory effect on genomic RNA replication of DHAV-3 in DEF cells and release of DHAV-3 from the cells.

## Discussion

The present paper describes the propagation of DHAV-3 in primary DEF cells. Based on the qPCR and IIF assays, both virulent and attenuated strains were shown to cause productive infections in DEF cells. However, DHAV-3 failed to produce CPE and plaques, distinct from DHAV-3 in DEL cells in which the virus could induce CPE [40]. These findings support the view that DHAV-3 can establish noncytocidal, productive infections in DEF cells. It has been shown previously that chicken embryo adapted DHAV-1 causes a cytocidal infection in DEF cells [27, 39]. Taken together, these investigations suggest that the effect of DHAV-1 and DHAV-3 infections on DEF cells is disparate. The detection of viral RNA in medium of infected cultures indicated that the noncytocidal DHAV-3 can be released from the infected cells. Previous studies have shown that noncytolytic viruses may be released from cell membranes, whose permeability can be modified by viral proteins such as nonstructural protein 2B [42, 43]. In addition, it has been proposed that release of noncytolytic viruses can be mediated by autophagy [44]. Further studies are needed to elucidate the mechanism of the release of DHAV-3 from infected cells.

In the investigation of the viral growth kinetics in DEF cells, both virulent and attenuated strains were shown to replicate effectively in DEF cells. We noted that the viral load of C-GY peaked more rapidly than that of YDF120 in the cell cultures (medium and cells) and medium, and that there were significant differences in viral loads between the virulent and attenuated groups in cell cultures at 12 and 48 hpi and in medium at 24 hpi. These findings suggest that the virulent strain can be replicated more rapidly and efficiently in DEF cells than the attenuated strain in early infection period. The difference in virulence may be responsible for the distinct replication efficiency between virulent and attenuated strains in early infection period.

The present study also describes the effect of serum on DHAV-3 replication in DEF cells. Based on the neutralization tests, FCS was shown to exert an inhibitory effect on both attenuated and virulent strains in DEF cells. On the basis of the TODA assays, it has been shown that FCS mainly inhibits DHAV-3 replication at two stages including co-inoculation and post-inoculation periods. The present observation was in agreement with previous findings of Chalmers and Woolcock [41], who have suggested that FCS cannot prevent entry of DHAV-1 into the cells by blocking receptor sites.

In further investigations into the mechanism of action of FCS against DHAV-3, the inhibitory effect was reflected at three steps of the DHAV-3 life cycle, including attachment virions to the cell surface, replication of genomic RNA, and release of viral particles. We noted that FCS cannot enter the interior of DEF cells. Therefore, we consider it likely that the inhibitory effect of FCS on replication of genomic RNA might be attributed to action on progeny virions released into the medium, which may prevent virus from infecting other uninfected cells.

The inhibitors in normal serum named as non-specific inhibitors to distinguish them from specific antibody has been studied by many researchers, while information on serum inhibitors has been controversy and confusing. To date, a generally accepted view is that the inhibitors were characterized as a complex of several proteins [45–47]. Based on the results of Chalmers and Woolcock [42], we can speculate that inhibitors of DHAV-1 may occur in normal animal sera as a complex with other inert materials. It should be noted that the inhibitors of DHAV-1 was only found in several mammal sera, which was consistent with host susceptibility [48, 49]. These observations may support the opinions that the presence of non-specific inhibitors in serum would be a factor in natural resistance to infection with DHAV and that the non-specific inhibitors may be employed as potent inhibitors in combating DHAV infection.

## Conclusions

In summary, we have demonstrated that both virulent and attenuated strains of DHAV-3 can establish a noncytocidal, productive infection in DEF cells. Furthermore, FCS displays an inhibitory effect on DHAV-3 replication, which is reflected at three steps of the DHAV-3 life cycle, including attachment, replication, and release. It is likely that the inhibitory effect might be attributed to action of a non-specific inhibitory factor present in FCS directly on the virus. These findings may provide new insights into the development of potential antiviral agents.

## Methods

### Viruses

Two DHAV-3 strains, namely C-GY and YDF120, were used in this study. C-GY was isolated from a 2-week-old dead duckling exhibiting signs and lesions typical of DVH. The virus was propagated in 10-day-old embryonated duck eggs [14]. YDF120 was the 120th passage of the DHAV-3 C-YDF isolate in specific pathogen-free (SPF) embryonated chicken eggs [50]. The viruses were stored at − 80 °C as embryo homogenates. The homogenates were clarified by centrifugation at 10,000 g for 10 min, followed by filtration through 0.22 μm Sterile Syringe Filters (Millipore, Billerica, MA, USA). Using 10-day-old duck embryos and 9-day-old chicken embryos, the titers of C-GY and YDF120 were determined to be 10^5.0^ and 10^4.5^ 50% egg lethal dose (ELD_50_) per 0.1 ml, respectively.

### Antiserum

Antiserum against DHAV-3 was produced in 4-week-old SPF chicks as described previously [14]. The chicks were inoculated intramuscularly two times at weekly intervals with the C-GY virus at the dose of 5 × 10^5^ ELD_50_ per bird. The antiserum was bled at 6 weeks of age and inactivated at 56 °C for 30 min.

### Cells

Primary DEF cells were prepared from 12-day-old Pekin duck embryos by a standard method [51], and maintained in growth medium consisting of DMEM (Macgene, Beijing, China) supplemented with 10% FCS (Corning, NY, USA), 100 U/ml penicillin, and 0.1 mg/ml streptomycin. The cells were incubated at 37 °C in 5% CO_2_ until use.

### Virus propagation

Confluent monolayers of DEF cells grown in T25 flasks were washed three times with DMEM, and each of DEF cultures was inoculated with 0.5 ml (10^4^ ELD_50_) of virus. After 1 h adsorption at 37 °C, the inoculum was removed, and maintenance medium consisting of DMEM supplemented with 2% CS (Solarbio, Beijing, China), 100 U/ml penicillin, and 0.1 mg/ml streptomycin was added. The cells together with medium were harvested after incubation at 37 °C for 3 days. Subsequently, the cells were lysed by three freeze-thaw cycles, and subjected to centrifugation at 10,000 g for 10 min. The cell-free supernatants were harvested and passaged for additional 9 times in DEF cells. Apart from investigation of CPE, growth of viruses in DEF cells were also determined by testing the supernatants from each passage using the qPCR assay as described below. The 5th passages in DEF cells of C-GY and YDF120 of DHAV-3 were used as inocula in subsequent experiments.

### Plague formation assay

DEF cells were prepared in 24-well plates, seeded at 2 × 10^5^ cells per well. When confluent, the cells were washed three times with DMEM. The virus was diluted in 10-fold steps to 10^− 6^, and 0.2 ml from each dilution was used to inoculate two of monolayers of DEF cells. After 1 h adsorption at 37 °C, the inoculum was removed, and the cells were washed three times with DMEM. Each of the wells was overlaid with 0.5 ml of agarose-maintenance medium overlay containing 1% agarose L.M.P (Macgene, Beijing, China). Following 72 h incubation at 37 °C in 5% CO_2_, the cells were fixed with 4% paraformaldehyde (Macgene, Beijing, China) for 1 h at room temperature. Subsequently, the overlay was removed, and the cells were stained with 0.2% crystal violet (Macgene, Beijing, China) for 30 min for visualization of plaques.

### IIF staining of virus-infected cells

DEF cells were prepared in 24-well plates and washed as described in plague formation assay. The cells were inoculated with DHAV-3 at a multiplicity of infection (MOI) corresponding to 1 copy/cell. After 1-h adsorption at 37 °C, the cells were washed three times with DMEM and replenished with 2% CS DMEM. Following incubation at 37 °C for 36 h, the medium was removed and the cells were washed three times with phosphate buffered saline (PBS). Subsequently, the cells were fixed with 0.3 ml of pre-cold ethanol for 15 min at room temperature. The ethanol was removed and the cells were washed three times. Each of monolayers was inoculated with 0.3 ml of a 100-fold dilution of antiserum against DHAV-3. After incubation at 37 °C for 1.5 h, the cells were washed three times, 5 min every time, and stained with 0.3 ml of a 50-fold dilution of FITC-conjugated goat anti-chicken IgG (BioDragon, Beijing, China). After further incubation at 37 °C for 1 h, the cells were washed again and examined using fluorescence microscopy (Olympus, Tokyo, Japan).

### Determination of kinetics of viral RNA replication and virus release

DEF cells were prepared and inoculated with DHAV-3 as described in IIF test. After adsorption at 37 °C for 1 h, the cells were washed three times with DMEM and replenished with 2% CS DMEM. Incubation at 37 °C was continued. To investigate the kinetics of viral RNA replication and virus release, infected cultures (medium and cells) and medium were sampled respectively at different time points pi, and viral loads in the samples were detected by using qPCR assay.

### Analysis on effect of serum on virus

This assay was designed to investigate the effect of FCS and CS on DHAV-3 C-GY and YDF120 strains. Each serum was diluted in DMEM to form three concentrations (4, 2, and 1%). DMEM served as a control. The serum from each concentration was mixed with an equal volume of virus (2 × 10^5^ copies) diluted in DMEM, and incubated at 37 °C for 1 h. After monolayers of DEF cells (2 × 10^5^ cells) were maintained in culture in a 24-well plate and washed three times with DMEM, the cells were inoculated with 0.2 ml of the serum-virus mixtures and incubated for 1 h at 37 °C for virus adsorption. Next, the cells were washed three times with DMEM and replenished with 2% CS DMEM. The samples (medium together with cells) were collected at 36 hpi for C-GY and 48 hpi for YDF120. Viral load in each sample was detected by the qPCR method.

### TODA assay

To investigate the mode of action of FCS on DHAV-3 replication, the TODA assay was performed as described previously with modifications [52]. DEF cells were prepared as described in IIF test. When confluent, the cells were washed thrice with DMEM.

For the pre-inoculation assay, DEF cells were pre-incubated with 0.2 ml of 2% FCS DMEM, using 2% CS DMEM as a control. After treatment at 37 °C for 1 h, the cells were washed thrice with DMEM to remove unbound serum residues, and inoculated with DHAV-3 at an MOI corresponding to 1 copy/cell. After adsorption at 37 °C for 1 h, the cells were washed thrice with DMEM and replenished with 0.5 ml of 2% CS DMEM.

For the co-inoculation assay, DHAV-3 was mixed with FCS to give a final concentration of 2% of serum. 0.2 ml of the FCS-virus mixture (MOI of 1 copy/cell) was used to inoculate monolayers of DEF cells. The CS-virus mixture served as a control. After adsorption at 37 °C for 1 h, the cells were washed thrice with EMEM and replenished with 0.5 ml of 2% CS DMEM.

For the post-inoculation assay, DEF cells were inoculated with DHAV-3 at an MOI corresponding to 1 copy/cell. After 1-h adsorption at 37 °C, the cells were washed thrice and divided into four groups: the first group was replenished with 0.5 ml of 2% FCS DMEM, the second group with 0.5 ml of 2% CS DMEM, and the third and fourth groups with 0.5 ml of serum-free DMEM. After incubation at 37 °C for 1 h, FCS and CS were added into the cells in the third and fourth groups respectively to give a final concentration of 2% of serum.

The cells were incubated further at 37 °C in 5% CO_2_. In all three assays, three monolayers of DEF cells were used in each inoculation. The samples (medium and cells) were collected at 24 hpi. Viral load in each sample was detected by using the DHAV-3 qPCR assay.

### Mechanism of action of FCS against DHAV-3

To gain insight into the mechanisms by which FCS exerted its anti-DHAV-3 activity in DEF cells, the effect of FCS on the steps of the DHAV-3 life cycle was examined as described previously [53].

Firstly, the effect of FCS on viral attachment to cell surface was investigated. Serum and virus were individually diluted with pre-cold DMEM and mixed together to give a final concentration of 2% of serum. DEF cells were prepared in 24-well plates, seeded at 2 × 10^5^ cells per well. When confluent, the cells were washed thrice with pre-cold DMEM, and inoculated with 0.2 ml of the serum-virus mixture (MOI of 1 copy/cell). After adsorption at 4 °C for 1 h, the cells were washed thrice with pre-cold DMEM and replenished with 0.2 ml of DMEM. After incubation at 37 °C for 1 h, DMEM was removed, and 0.5 ml of 2% CS DMEM was added. Incubation was continued at 37 °C in 5% CO_2_ and samples (medium and cells) were collected at 24 hpi.

Secondly, the effect of FCS on virus entry into cells was investigated. DEF cells were prepared and washed as described above. The cells were inoculated with DHAV-3 at an MOI of 1 copy/cell. Following 1 h adsorption at 4 °C, inoculum was removed, and the cells were washed thrice with pre-cold DMEM to remove unbound viruses. The cells were treated with 0.2 ml of 2% FCS DMEM, using 2% CS DMEM as a control. After treatment at 37 °C for 1 h, the maintenance medium was removed and the cells were washed thrice with DMEM. The cells were then replenished with 0.5 ml of 2% CS DMEM and incubated further at 37 °C in 5% CO_2_. Samples (medium and cells) were collected at 24 hpi.

Finally, the effect of FCS on viral RNA replication and virus release was assessed. The experiments were performed as described in post-inoculation assay. The samples were collected at 36 hpi for C-GY and 48 hpi for YDF120. For assessing viral RNA replication, medium and cells were sampled. For virus release assay, medium was sampled.

In all experiments, each group contained three replications. Viral load in each sample was quantified as described below.

### Virus quantification

DHAV-3 loads in samples collected from infected DEF cells were quantified by using a DHAV-3 qPCR assay. Briefly, forward primer DHAV-3/F1 (5′-TGACCCACGTTTAAGTCTCTATG-3′) and reverse primer DHAV-3/R1 (5′-CTCGGCACAGGATCCAATAATC-3′) were designed on the basis of the genome sequence of C-YDF (GenBank accession no. GU066821). The primers were applied in a conventional reverse transcription (RT)-PCR assay to amplify a 515-bp product from the 5′UTR–VP0 region of the YDF120 genome. The reaction mixture and condition were the same as described previously [14]. The PCR product was cloned into the pCloneEZ-Blunt-AMP/HC (Taihegene, Beijing, China), resulting in a recombinant plasmid pC-YDF120.

The concentration of plasmid pC-YDF120 was measured using Biodropsis BD-1000 ultraviolet spectrophotometry (Beijing Oriental Science and Technology Development, Beijing, China). Standard curve for the qPCR assay was generated using 2 μl from each of 10-fold serial dilutions (10^− 3^–10^− 7^; corresponding to 3.83 × 10^5^–38.3 copies/μl) of vector construct pC-YDF120 as templates. qPCR was performed using forward primer DHAV-3/F2(5′-TGGTCGAGTCCCATACACTATAA-3′) and reverse primer DHAV-3/R1, which were used to amplify a 106 bp sequence from the 515 bp 5′UTR-VP0 region of DHAV-3. The reaction mixture and condition were provided by AceQ qPCR SYBR Green Master Mix Kit (Vazyme, Nanjing, China).

For determination of viral load, the samples (medium or medium together with cells) were subjected to three frozen-thaw cycles, followed by centrifugation at 10000 g for 10 min. RNA was extracted from 200 μl supernatant using a TRIzol reagent (Thermo Scientific, Waltham, MA, USA), and diluted in 50 μl of RNase-free water. 5 μl of RNA was reverse transcribed into cDNA using a HiScript first Strand cDNA Synthesis Kit and random hexamers (Vazyme, Nanjing, China). 2 μl of cDNA was quantified using qPCR.

### Statistical analysis

Data were calculated as means ± standard deviation (SD). Differences between groups were analyzed using an independent samples t-test implemented in the SPSS Statistics 21th software (IBM, Armonk, NY, USA). A *P* < 0.05 value was considered statistically significant.

## Abbreviations

CPE: Cytopathic effect
CS: Chick serum
DEF: Duck embryo fibroblast
DEK: Duck embryo kidney
DEL: Duck embryo liver
DHAV-1: Duck hepatitis A virus genotype 1
DHAV-2: Duck hepatitis A virus genotype 2
DHAV-3: Duck hepatitis A virus genotype 3
DHV-2: Duck hepatitis virus type 2
DHV-3: Duck hepatitis virus type 3
DMEM: Dulbecco’s modified Eagle’s medium
DPI: Days post inoculation
DVH: Duck viral hepatitis
ELD_50_: 50% egg lethal dose
FCS: Fetal calf serum
FITC: Fluorescein isothiocyanate
HPI: Hours post inoculation
IIF: Indirect immunofluorescence
MOI: Multiplicity of infection
NT: nucleotides
ORF: Open reading frame
PBS: Phosphate buffered saline
qPCR: Quantitative real-time PCR
RT: Reverse transcription
SD: Standard deviation
SPF: Specific pathogen-free
TODA: Time-of-drug-addition
UTR: Untranslated region

## References

[1] Woolcock PR, Saif YM, Barnes HJ, Glisson JR, Fadly AM, McDougald LR, Swayne DE (2003). Duck hepatitis. Diseases of poultry, 11th Edn. Ames: Iowa state press.

[2] Levine PP, Fabrican J (1950). A hitherto-undescribed virus disease of ducks in North America. Cornell Vet.

[3] Tseng CH, Tsai HJ (2007). Molecular characterization of a new serotype of duck hepatitis virus. Virus Res.

[4] Kim MC, Kwon YK, Joh SJ, Kim SJ, Tolf C, Kim JH, Sung HW, Lindberg AM, Kwon JH (2007). Recent Korean isolates of duck hepatitis virus reveal the presence of a new geno- and serotype when compared to duck hepatitis virus type 1 type strains. Arch Virol.

[5] Knowles NJ, Hovi T, Hyypiä T, King AMQ, Lindberg AM, Pallansch MA, Palmenberg AC, Simmonds P, Skern T, Stanway G, Yamashita T, Zell R, King AMQ, Adams MJ, Carstens EB, Lefkowitz EJ (2011). Picornaviridae. Virus taxonomy. Classification and nomenclature of viruses: ninth report of the international committee on the taxonomy of viruses.

[6] Asplin FD (1965). Duck hepatitis: vaccination against two serological types. Vet Rec.

[7] Gough RE, Collins MS, Borland E, Keymer LF (1984). Astrovirus-like particles associated with hepatitis in ducklings. Vet Rec..

[8] Gough RE, Borland ED, Keymer IF, Stuart JC (1985). An outbreak of duck hepatitis type II in commercial ducks. Avian Pathol..

[9] Haider SA, Calnek BW (1979). In vitro isolation, propagation and characterization of duck hepatitis virus type III. Avian Dis.

[10] Bosch A, Guix S, Krishna NK, Méndez E, Monroe SS, Pantin-Jackwood M, Schultz-Cherry S, King AMQ, Adams MJ, Carstens EB, Lefkowitz EJ (2011). Astroviridae. Virus taxonomy. Classification and nomenclature of viruses: ninth report of the international committee on the taxonomy of viruses.

[11] Todd D, Smyth VJ, Ball NW, Donnelly BM, Wylie M, Knowles NJ, Adair BM (2009). Identification of chicken enterovirus-like viruses, duck hepatitis virus type 2 and duck hepatitis virus type 3 as astroviruses. Avian Pathol..

[12] Pan M (2011). Construction of an infectious cDNA clone of duck hepatitis a virus type 3 and functional analysis of its 5′UTR.

[13] Toth TE (1969). Studies of an agent causing mortality among ducklings immune to duck virus hepatitis. Avian Dis.

[14] Fu Y, Pan M, Wang X, Xu Y, Yang H, Zhang D (2008). Molecular detection and typing of duck hepatitis a virus directly from clinical specimens. Vet Microbiol.

[15] Doan HT, Le XT, Do RT, Hoang CT, Nguyen KT, Le TH (2016). Molecular genotyping of duck hepatitis a viruses (DHAV) in Vietnam. J Infect Dev Ctries.

[16] Lin SL, Cong RC, Zhang RH, Chen JH, Xia LL, Xie ZJ, Wang Y, Zhu YL, Jiang SJ (2016). Circulation and in vivo distribution of duck hepatitis a virus types 1 and 3 in infected ducklings. Arch Virol.

[17] Soliman M, Alfajaro MM, Lee MH, Jeong YJ, Kim DS, Son KY, Kwon J, Choi JS, Lim JS, Choi JS, Lee TU, Cho KO, Kang MI (2015). The prevalence of duck hepatitis a virus types 1 and 3 on Korean duck farms. Arch Virol.

[18] Zhang D, Liu N, Liu D (2016). Duck hepatitis virus. Molecular detection of animal viral pathogens.

[19] Ding C, Zhang D (2007). Molecular analysis of duck hepatitis virus type 1. Virology..

[20] Tseng CH, Knowles NJ, Tsai HJ (2007). Molecular analysis of duck hepatitis virus type 1 indicates that it should be assigned to a new genus. Virus Res.

[21] Kim MC, Kwon YK, Joh SJ, Lindberg AM, Kwon JH, Kim JH, Kim SJ (2006). Molecular analysis of duck hepatitis virus type 1 reveals a novel lineage close to the genus *Parechovirus* in the family *Picornaviridae*. J Gen Virol.

[22] Wang L, Pan M, Fu Y, Zhang D (2008). Classification of duck hepatitis virus into three genotypes based on molecular evolutionary analysis. Virus Genes.

[23] Akulov AV, Kontrimavichus LM, Maiboroda AD (1972). Susceptibility of geese to duck hepatitis virus. Veterinariya.

[24] Davis D, Woolcock PR (1986). Passage of duck hepatitis virus in cell cultures derived from avian embryos of different species. Res Vet Sci.

[25] Fitzgerald JE, Hanson LE, Wingard M (1963). Cytopathic effects of duck hepatitis virus in duck embryo kidney cell cultures. Proc Soc Exp Biol Med.

[26] Fu Y, Chen Z, Li C, Liu G (2012). Establishment of a duck cell line susceptible to duck hepatitis virus type 1. J Virol Methods.

[27] Golubnichi VP, Tishchenko GP, Korolkov VI (1976). Preparation of tissue culture antigens of duck hepatitis virus. Vet NaukProizTr.

[28] Hwang J (1965). Duck hepatitis virus in duck embryo fibroblast cultures. Avian Dis.

[29] Hwang J (1966). Duck hepatitis virus in duck embryo liver cell cultures. Avian Dis.

[30] Kaeberle ML, Drake JW, Hanson LE (1961). Cultivation of duck hepatitis virus in tissue culture. Proc Soc Exp Biol Med.

[31] Kaleta EF (1988). Duck viral hepatitis type 1 vaccination: monitoring of the immune response with a microneutralisation test in Pekin duck embryo kidney cell cultures. Avian Pathol.

[32] Maiboroda AD (1972). Formation of duck hepatitis virus in culture cells. Veterinariya..

[33] Mészáros I, Tóth R, Bálint A, Dán A, Jordan I, Zádori Z (2014). Propagation of viruses infecting waterfowl on continuous cell lines of Muscovy duck (*Cairina moschata*) origin. Avian Pathol..

[34] Pollard M, Starr TJ (1959). Propagation of duck hepatitis virus in tissue culture. Proc Soc Exp Biol Med.

[35] Pollard M, Starr TJ (1960). Propagation of duck hepatitis virus in tissue cultures prepared with collagenase. J Bacteriol.

[36] Wang W, Said A, Wang Y, Fu Q, Xiao Y, Lv S, Shen Z (2016). Establishment and characterization of duck embryo epithelial (DEE) cell line and its use as a new approach toward DHAV-1 propagation and vaccine development. Virus Res.

[37] Yao F, Chen Y, Shi J, Ming K, Liu J, Xiong W, Song M, Du H, Wang Y, Zhang S (2016). Wu 1, Wang D, Hu Y. replication cycle of duck hepatitis a virus type 1 in duck embryonic hepatocytes. Virology.

[38] Woolcock PR, Chalmers WS, Davis D (1982). A plaque assay for duck hepatitis virus. Avian Pathol.

[39] Woolcock PR (1986). An assay for duck hepatitis virus type I in duck embryo liver cells and a comparison with other assays. Avian Pathol.

[40] Zhang B, Zhang HR, Yang FL, Tang C, Yue H (2013). Proliferation of duck hepatitis a virus genotype C in duck embryo liver cells. Chinese Veterinary Science.

[41] Chalmers WS, Woolcock PR (1984). The effect of animal sera on duck hepatitis virus. Avian Pathol.

[42] Nieva JL, Agirre A, Nir S, Carrasco L (2003). Mechanisms of membrane permeabilization by picornavirus 2B viroporin. FEBS Lett.

[43] van Kuppeveld FJ, Hoenderop JG, Smeets RL, Willems PH, Dijkman HB, Galama JM, Melchers WJ (1997). Coxsackievirus protein 2B modifies endoplasmic reticulum membrane and plasma membrane permeability and facilitates virus release. EMBO J.

[44] Taylor MP, Burgon TB, Kirkegaard K, Jackson WT (2009). Role of microtubules in extracellular release of poliovirus. J Virol.

[45] Križanová O, Rathová V (1969). Serum inhibitors of myxoviruses. Curr Top Microbiol Immunol.

[46] Beisner B, Kool D, Marich A, Holmes IH (1998). Characterisation of G serotype dependent non-antibody inhibitors of rotavirus in normal mouse serum. Arch Virol.

[47] Job ER, Bottazzi B, Gilbertson B, Edenborough KM, Brown LE, Mantovani A, Brooks AG, Reading PC (2013). Serum amyloid P is a sialylated glycoprotein inhibitor of influenza a viruses. PLoS One.

[48] Hwang J. Susceptibility of poultry to duck hepatitis viral infection. Am J Vet Res. 1974.

[49] Reuss U (1959). Virusbiologische untersuchungen bei der Entenhepatitis. Zentralblatt für Veterinärmedizin.

[50] Yuan Y (2013). Development of the original seeds for an attenuated vaccine of duck hepatitis a virus 3. M.S. dissertation.

[51] Hernandez R, Brown DT (2010). Growth and maintenance of chick embryo fibroblasts (CEF). Curr Protoc Microbiol.

[52] Daelemans D, Pauwels R, De Clercq E, Pannecouque C (2011). A time-of–drug addition approach to target identification of antiviral compounds. Nat Protoc.

[53] Tan CW, Sam IC, Chong WL, Lee VS, Chan YF (2017). Polysulfonate suramin inhibits Zika virus infection. Antivir Res.

